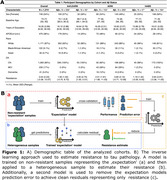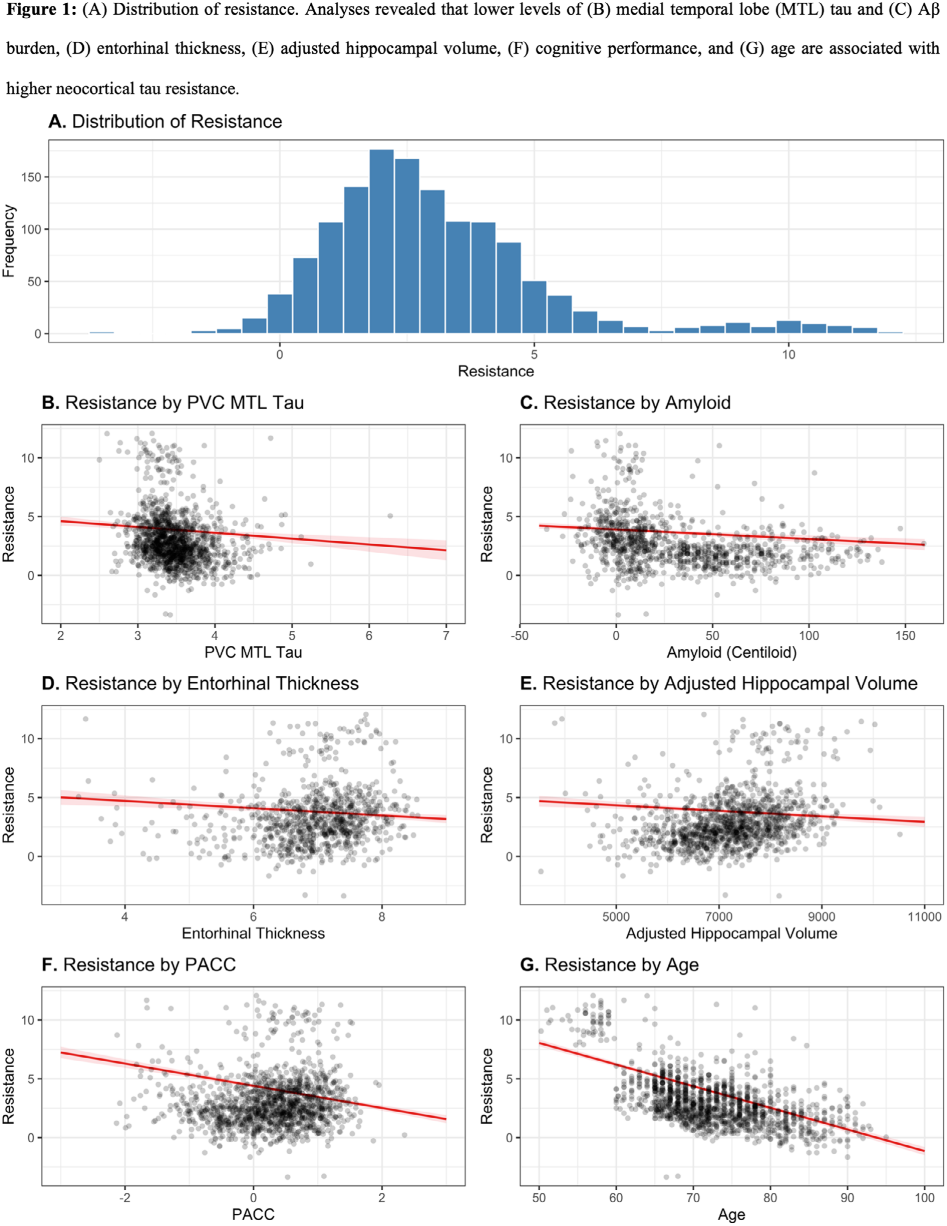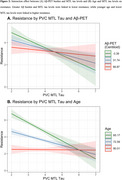# Biomarker and cognitive associates of resistance to neocortical tau pathology in a pooled cohort

**DOI:** 10.1002/alz70857_106460

**Published:** 2025-12-25

**Authors:** Colin Birkenbihl, Hannah M Klinger, Michael J. Properzi, Madison Cuppels, Jane A Brown, Gillian T Coughlan, Mabel Seto, Annie Li, Jasmeer P. Chhatwal, Bernard J Hanseeuw, Michelle E. Farrell, Heidi I.L. Jacobs, Maria Carrigan, Oliver Langford, Julie C Price, Aaron P. Schultz, Dorene M. Rentz, Rebecca E. Amariglio, Keith A. Johnson, Reisa A. Sperling, Michael C. Donohue, Timothy J. Hohman, Rachel F. Buckley

**Affiliations:** ^1^ Massachusetts General Hospital, Harvard Medical School, Boston, MA, USA; ^2^ Department of Neurology, Massachusetts General Hospital, Harvard Medical School, Boston, MA, USA; ^3^ Brigham and Women's Hospital, Harvard Medical School, Boston, MA, USA; ^4^ Athinoula A. Martinos Center for Biomedical Imaging, Massachusetts General Hospital and Harvard Medical School, Boston, MA, USA; ^5^ Faculty of Science, Swammerdam Institute for Life Sciences, University of Amsterdam, Amsterdam, North Holland, Netherlands; ^6^ Alzheimer's Therapeutic Research Institute, University of Southern California, San Diego, CA, USA; ^7^ Department of Neurology, Vanderbilt Memory & Alzheimer's Center, Vanderbilt University Medical Center, Nashville, TN, USA

## Abstract

**Background:**

Prior evidence suggests that neocortical tau in those with higher β‐amyloid (Aβ) may be the main driver of Alzheimer's disease (AD)‐related neurodegeneration leading to insidious cognitive decline and ultimately a diagnosis of AD dementia. Resistance to the ‘spread’ of neocortical tau pathology from localized medial temporal (MTL) regions can be defined as individuals having lower neocortical tau than expected given their individual characteristics, such as demographics and Aβ burden. We examined associations between resistance to neocortical tau pathology and various markers of AD pathology, cognitive performance, and brain reserve.

**Method:**

We calculated tau resistance using our published inverse learning method (Figure 1B), which estimates the deviation away from a model trained on an expectation sample (278 Aβ‐PET+ older adults with high neocortical tau‐PET (PVC_SUVR_composite_:inferior temporal/inferior parietal/fusiform/middle temporal) burden based on Gaussian Mixture Modeling;Figure 2A). We ran a series of linear regression models on the remaining 1,374 older adults pooled from the Harvard Aging Brain Study (HABS), ADNI, and A4/LEARN (Demographics in Figure 1A). We examined associations between tau resistance and 1) MTL tau‐PET (PVC_SUVR_composite_; entorhinal/amygdala/parahippocampal), 2) measures of brain reserve (hippocampal volume and entorhinal cortical thickness), 3) neocortical Aβ‐PET burden (Centiloids), 4) an interaction between MTL tau and Aβ‐PET, and 5) cognitive performance (PACC). All models adjusted for age, sex, education, cohort, and *APOE*ε4.

**Results:**

Lower MTL tau, lower Aβ, and younger age were significantly associated with higher neocortical tau resistance (β_MTL_=‐0.22(0.06), *p* < 0.001, Figure 2B; β_Aβ_=‐0.13(0.03), *p* <0.001Figure 2C;β_Age_=‐0.62(0.02), *p* < 0.001). Higher PACC, greater hippocampal volume and thicker entorhinal cortices were associated with lower resistance (β_PACC_=‐0.29(0.02), *p* < 0.001, Figure 2D;β_HV_=‐0.10(0.03), *p* < 0.001, β_EC_thickness_=‐0.14(0.03), *p* < 0.001). Greater Aβ and MTL tau burden interacted to influence lower resistance (Figure 3). In a sample limited to Aβ+ (*N* = 537), we found only lower PACC and younger age significantly associated with tau resistance.

**Conclusion:**

These findings suggest that baseline levels of MTL tau and age play a role in resisting the advancement of tauopathy into neocortical brain regions, and might be mediated by Aβ in early disease stages. The counter‐intuitive association with cognition and brain reserve measures implies that tau resistance is most likely represented by those with greater cognitive impairment and lower reserve, as neocortical tau burden is much lower in clinically‐normal older adults.